# Impaired Fine Motor Function of the Asymptomatic Hand in Unilateral Parkinson’s Disease

**DOI:** 10.3389/fnagi.2019.00266

**Published:** 2019-10-04

**Authors:** Xiaojuan Dan, Jia Liu, Julien Doyon, Yongtao Zhou, Jinghong Ma, Piu Chan

**Affiliations:** ^1^Department of Neurology and Neurobiology, Xuanwu Hospital of Capital Medical University, Beijing, China; ^2^Key Laboratory on Neurodegenerative Disorders of Ministry of Education, Key Laboratory on Parkinson’s Disease of Beijing, Beijing, China; ^3^Department of Geriatrics, Xuanwu Hospital of Capital Medical University, Beijing, China; ^4^McConnell Brain Imaging Center, Department of Neurology and Neurosurgery, Montreal Neurological Institute, McGill University, Montreal, QC, Canada; ^5^National Clinical Research Center for Geriatric Disorders, Beijing, China; ^6^Beijing Institute for Brain Disorders Parkinson’s Disease Center, Advanced Innovation Center for Human Brain Protection, Capital Medical University, Beijing, China

**Keywords:** Parkinson’s disease, quantitative evaluation, early diagnosis, disease progression, dual-task effect

## Abstract

The early detection of Parkinson’s disease (PD) still remains a challenge to date. Although studies have previously reported subtle motor function abnormalities in early PD patients, it is unclear whether such clinical signs can be better detected while patients are concurrently performing a cognitive task, and whether they can be useful in predicting patients’ clinical conversion state. Seventy-two right-handed participants (40 drug-naive patients with idiopathic unilateral PD and 32 age-matched healthy controls) were enrolled in this study. All participants were asked to perform the Purdue Pegboard test (PPT) either alone (single-task condition) or during a concurrent mental subtraction-by-3 task (dual-task condition). A 4-year telephone follow-up was later conducted to determine whether PD patients converted to bilateral signs. We found that PD patients showed a significant reduction in dexterity on the PPT compared to the controls in both single- and dual-task conditions. Yet patients’ performance in the dual-task condition revealed a greater interference effect when patients performed the task with their right hand than with their left hand. PPT also revealed reasonable discriminative ability for prediagnosing PD. However, dual-tasking did not have added value in differentiating early patients and controls. At follow-up, the baseline PPT performance of the asymptomatic hands was positively correlated with time to convert from unilaterally to bilaterally affected states (*r* = 0.62, *P* = 0.031). Together, these findings suggest that PPT can serve as a useful auxiliary tool in evaluating early PD, and shed light on the neuroplasticity mechanism of fine motor deficit at this very early stage.

## Introduction

Parkinson’s disease (PD) is the second most common neurodegenerative disorder, which is characterized pathologically by the progressive loss of dopaminergic neurons in the substantia nigra, and clinically by the presence of cardinal motor symptoms including bradykinesia, resting tremor and/or rigidity. Yet despite major advances in our understanding of this disease, biomarkers that reflect the onset of PD’s pathology, leading to its early diagnosis, are still lacking. Consequently, diagnosing PD early on the prodromal phase, during which putative neuroprotective or disease-modulating therapies might be more effective, remains an unsolved challenge (Lang, 2011; Berg et al., 2015). Typically at onset, the clinical motor deficits in PD are unilateral, and this phenomenon can last for several months to years before clinical symptoms become bilateral (Zhao et al., 2010; Wang et al., 2015). Postmortem and imaging studies have shown that the asymmetry in clinical motor deficits observed in the early stage of the disease reflect the known asymmetry in pathological progression of loss of dopaminergic neurons in the two hemispheres (Kempster et al., 1989; Morrish et al., 1995; Rinne et al., 2001; Kumakura et al., 2010; Wang et al., 2015). Thus, one can assume that the assessment of motor functions on the asymptomatic side of an unilaterally-affected PD patients provides a “model/proxy” of prodromal PD, which can be useful to identify potential biomarkers in the early phase of this neurological condition.

Among the motor deficits often seen in PD, patients frequently report difficulties with hands’ dexterity (Nijkrake et al., 2009). Traditionally manual dexterity has been evaluated using the Purdue pegboard test (PPT; Blair et al., 1987; Nozaki et al., 2018); a simple and inexpensive quantitative assessment designed to measure fine motor functions with good test-retest reliability (Lee et al., 2013). In fact, previous studies have shown that PPT is a more objective and sensitive measure of disease severity than the most commonly used clinical measurement, i.e., the Unified Parkinson’s Disease Rating Scales (UPDRS) part III (Haaxma et al., 2008). Performance on PPT has thus frequently been used to assess the effects of medication and neurosurgery interventions on dexterity in patients with PD (Fregni et al., 2006; Slowinski et al., 2007; Espay et al., 2015). Furthermore, compared to seven other motor function tests, performance on the PPT has resulted in the best receiver operating characteristic (ROC) curve for patients vs. age-matched controls and yielded reasonable accuracy to detect subtle motor dysfunctions in PD patients (Haaxma et al., 2010).

Importantly, recent studies have reported that difficulty in performing two tasks simultaneously (i.e., dual-task condition) may also be a sensitive indicator of early PD impairment (Fuller et al., 2013). For instance, it is well known that talking while walking causes gait deterioration in PD patients. Previous studies have investigated concurrent performance on a cognitive task while PD patients were carrying on the PPT task, and demonstrated a greater extent of dual-task interference in this group of patients. However, it remains unclear whether PPT performance can be sensitive to detect motor impairment in the prodromal stage of PD and/or when administered in a dual-task interference paradigm. In the present study, we thus used performance on the clinically unaffected side of patients with unilateral symptoms as a proxy of “prodromal” PD, and sought to investigate whether PPT can serve as a potential auxiliary tool in detecting early disease state and monitoring disease progression in “prodromal” PD patients. Specifically, drug-naive patients with clinically ascertained, unilaterally affected PD were compared to age and gender-matched normal control subjects for their manual dexterity between: (1) the symptomatic hand and the corresponding hand of normal controls; as well as (2) the asymptomatic hand and the corresponding hand of normal controls; and for (3) the dual-task interference during concurrent cognitive performance. In addition, we conducted a 4-year telephone follow-up to determine whether the level of dexterity of the asymptomatic hand could be an efficient predictor of a faster development from a clinically-unilateral to bilaterally-affected condition in this group of patients.

## Materials and Methods

### Subjects

To control for and minimize the effects of medication involvement and disease severity, only clinically ascertained, unilaterally affected *de novo*, drug-naive PD patients were recruited for this study. Forty idiopathic PD patients with unilateral clinical signs, including 16 left-onset (PD-L, 47–71 years of age) and 24 right-onset (PD-R, 42–73.3 years of age) patients, as well as 32 age-matched (43–75 years of age) healthy controls, participated in this study. They were recruited from the Parkinson and Movement Disorder Center at Xuanwu Hospital of Capital Medical University in Beijing. PD diagnosis was made by movement disorders specialists, following the United Kingdom PD Society Brain Bank Criteria. The evaluation of UPDRS III motor score was conducted by two movement disorders specialists who were blind to the clinical information of the patients. Only unilateral cases with no clinical signs on the contralateral (less affected) side were recruited in the study. Patients with a positive family history of PD, secondary parkinsonism, or other forms of atypical parkinsonism were excluded. Age and gender-matched healthy controls were recruited from the Beijing Longitudinal Study on Aging community cohort. Hand dominance was determined using the Edinburgh Handedness Inventory. All subjects were right-handed and of Chinese Han ethnicity. Participants classified as musicians or professional typists were excluded. Subjects with a Mini-Mental State Examination (MMSE) score of <24 or a Geriatric Depression Scale-30 items (GDS-30) score of >10 were also excluded. This study was approved by the Ethics Committee of Xuanwu Hospital of Capital Medical University. All participants gave written informed consent before inclusion in the study.

### Purdue Pegboard Test Tasks

All participants performed the PPT (Model 32020, Lafayette, IN, USA) under both single-task and dual-task conditions (Supplementary Figure S1). In the single condition, the PPT consisted of performing the task with the dominant right hand (DRH) and the non-dominant left hand (NDLH) in randomized order. Briefly, subjects were instructed to transfer a series of small metal rods, from the outer concave cup located on the same side of the hand used, one at a time into corresponding holes of the board as quickly as possible within 30 s. Participants were given the opportunity to practice for 10 s before the timed tests began to ensure that participants understood the instructions. Each hand was tested three times (trial) in a row. The mean number of pegs placed in three trials was scored for each hand.

In order to test participants’ abilities to perform the PPT in a dual-task condition, we then administered simultaneously a verbal cognitive task requiring participants to subtract 3 as quickly and accurately as possible within 30 s from a number randomly selected from 280 to 320. The cognitive task condition alone and the dual-task condition were also administered on three occasions in a row. For each trial, the cognitive task was administered with different numbers. Subjects were instructed to try not to concentrate on one task in particular, but to perform both tasks at the same time. Both the number of subtractions and the number of pegs transferred during each test were then recorded. The order of administration of the motor test alone, the cognitive test alone and the dual-test condition was randomized. All the tests were administered in the morning and in a room at constant temperature (Muller et al., 2011).

### Clinical and Neuropsychological Assessment

Two movement disorder specialists conducted the standard clinical evaluations, including the UPDRS and the Hoehn & Yahr (H&Y) staging of the PD patients. The telephone follow-up was conducted every half a year for 4 years. Global cognitive functioning was assessed using the MMSE. Additional and more detailed neuropsychological evaluations included tests of working memory (digit span forward and backwards), visuospatial abilities (Stereo acuity, clock-drawing test) and attention/executive functions (Stroop color-word test, Trail Making test part A and part B).

### Statistical Analysis

The participants’ demographics, results of the neuropsychological examination and the groups’ performance on PPT as well as on the number of serial subtractions under single-task or dual-task conditions, were compared. Normal distribution tests were performed before each of the continuous variable comparisons. Group differences were analyzed using the Pearson’s *χ*^2^ test for categorical variables and the Student’s *t*-test or analysis of variance (ANOVA) for continuous variables with normal distribution, and Mann–Whitney *U* test or Kruskal–Wallis *H* test for non-normal distributed variables. The participants’ PPT scores showed a normal distribution during both single-task and dual-task conditions (Supplementary Table S1). Further analyses were performed using the general linear model, with age and gender adjustment, and included main effects of group and condition, as well as a group-by-condition interaction term, to account for the dependency among observations (i.e., the 3-subtraction and PPT performance). The significant main effect of group, condition, or interaction differences were compared with *post hoc* analyses.

To investigate the dual-task effect on both cognitive and motor performances as reported in previous studies (Bock, 2008), relative changes in performances between the dual- and single-task conditions were calculated to get a measure of interference using the following equation: [condition 2 (dual-task) − condition 1 (single-task)]/condition 1 (single-task) × 100 = interference effect, with a negative value indicating a reduction in speed during the dual-task (i.e., the larger the negative value, the greater the interference effect).

ROC curves were drawn to visualize the discriminative ability of PPT performance; areas under the curve (AUC), as well as the highest sensitivity and specificity, were calculated. We further used standard linear regressions to explore associations between scores on clinical motor assessment scales and PPT performance, while at the same time adjusting for age, gender and disease duration.

At follow-up, the asymptomatic hand pegboard dexterity performance at baseline was compared between patients who remained unilaterally affected vs. those who converted to a bilateral condition during the 4-year period. Furthermore, correlations between the patients’ asymptomatic hand PPT performance and the time interval for clinical conversion were calculated to test whether performance at baseline could predict the patients’ clinical profile 4 years later. All statistical analyses were carried out using SPSS software for windows version 21.0. The statistical significance threshold was set at a 2-tailed *p*-value < 0.05. Bonferroni corrections were also applied for *post hoc* analyses.

## Results

### Demographics

Demographics, neuropsychological tests results, and clinical characteristics of the study participants are given in Table 1. Based on the side of clinical symptoms onset, PD patients were divided into two groups: Right hand-onset (PD-R, i.e., the DRH was symptomatic while the NDLH was asymptomatic) or Left hand-onset (PD-L, i.e., the NDLH was symptomatic and the DRH was asymptomatic). Scores based on activities of daily living, as measured with the UPDRS part II, were inferior in patients with PD-R compared to the PD-L (*P* = 0.038) group, although the level of motor impairment assessed with the UPDRS part III was comparable between the PD-R (6.71 ± 1.76) and PD-L (6.94 ± 3.09) groups, hence suggesting that patients who were clinically affected on the dominant side had lower level of activities of daily living than those who experienced motor deficits on the non-dominant side.

**Table 1:** Demographic and disease characteristics of the subjects.

	PD-R	PD-L	Control	*P^a^	*P^b^	*P^c^	*P^d^
**Demographics**
*N*	24	16	32
Gender, male, *n* (%)	12 (50.0)	10 (62.5)	15 (46.9)	0.586	0.436	0.307	0.817
Age (years)	57.57 ± 8.53	58.77 ± 6.78	60.52 ± 8.11	0.388	0.645	0.475	0.176
Education (years)	11.17 ± 2.67	10.69 ± 3.09	10.94 ± 2.86	0.973	0.811	0.920	0.859
Disease duration (years)	1.80 ± 1.17	1.15 ± 0.75			0.056		
GDS-30	6.79 ± 3.13	6.31 ± 3.20	4.31 ± 3.85	0.017	0.726	0.041	0.009
MMSE	28.58 ± 1.35	28.19 ± 1.94	29.16 ± 0.95	0.157	0.733	0.109	0.111
**Neuropsychological tests**
TMT _A_	67.04 ± 27.17	66.60 ± 18.20	54.34 ± 19.09	0.089	0.795	0.055	0.085
TMT _B_	137.62 ± 74.56	130.73 ± 53.17	113.47 ± 41.36	0.447	0.965	0.386	0.236
TMT _B-A_	64.13 ± 43.06	70.58 ± 60.83	59.12 ± 30.66	0.998	0.908	0.927	0.987
Stroop1	23.96 ± 4.98	24.50 ± 3.92	23.66 ± 5.26	0.624	0.479	0.317	0.960
Stroop2	31.52 ± 7.23	29.88 ± 4.56	29.47 ± 5.08	0.741	0.786	0.801	0.412
Stroop interference	53.61 ± 13.30	54.56 ± 12.37	50.78 ± 14.79	0.344	0.943	0.158	0.306
Digit-span (forward)	8.00 ± 0.89	8.08 ± 1.12	8.20 ± 0.93	0.721	0.908	0.486	0.521
Digit-span (backward)	4.14 ± 1.06	4.23 ± 0.93	4.70 ± 1.56	0.521	0.684	0.515	0.282
Stereo acuity	6.00 ± 2.71	6.69 ± 2.24	7.37 ± 2.46	0.142	0.471	0.244	0.061
Clock copy	2.79 ± 0.51	2.88 ± 0.34	2.91 ± 0.29	0.693	0.688	0.741	0.394
**Clinical evaluation**
UPDRS part I	0.88 ± 1.23	0.44 ± 0.51			0.366
UPDRS part II	7.04 ± 2.33	5.38 ± 2.50			0.017
UPDRS part III	10.33 ± 3.06	9.81 ± 4.04			0.587
UPDRS total scores	15.62 ± 6.38	18.25 ± 5.12			0.070
Hoehn & Yahr	1.19 ± 0.32	1.19 ± 0.31			0.932
^e^Tremor	1.92 ± 1.47	2.94 ± 1.61			0.043
^f^Bradykinesia	4.42 ± 2.15	3.38 ± 1.59			0.109
^g^Rigidity	2.08 ± 1.41	1.81 ± 0.91			0.487
Bulbar abnormalities	1.42 ± 0.78	1.38 ± 0.89			0.905
L-UPDRSIII scores	0 ± 0	6.94 ± 3.09
R-UPDRSIII scores	6.71 ± 1.76	0 ± 0

PD-R, right-onset Parkinson’s disease; PD-L, left-onset Parkinson’s disease; GDS-30, the 30 items Geriatric Depression Scale; MMSE, Mini-Mental Status Examination; TMT, Trail Making Test; UPDRS, Unified Parkinson’s Disease Rating Scale; H&Y, Hoehn & Yahr; p^a^, p-value among the three groups: PD-L, PD-R, and normal control; p^b^, p-value between PD-L and PD-R; p^c^, p-value between PD-L and normal control; p^d^, p-value between PD-R and normal control. *p-value was calculated using Pearson’s *χ*^2^ test for categorical variables, student’s *t*-test or analysis of variance for continuous variables with normal distribution, and Mann–Whitney U test or Kruskal–Wallis H test for non-normal distribution variables. ^e^Score was the sum of item 20 and 21 in the UPDRS. ^f^Score was the sum of item 23, 24 and 25 in the UPDRS. ^g^Score was the sum of item 22 in the UPDRS.

### Performance on the PPT and 3-Subtraction Task: Single-Task Condition

As reported in Table 2 and as expected, the level of hand dexterity was lower in the symptomatic hand of PD patients as compared to the corresponding hand of healthy controls. Importantly, PPT performance using the asymptomatic hand in both PD groups was also significantly impaired as compared to the healthy controls (PD-R, *P* < 0.001; PD-L, *P* = 0.01). By contrast, there was no significant difference in performance on the 3-subtraction task among the three groups.

**Table 2:** Uni- and dual-tasks of subtract 3 and Purdue Pegboard performance in the hemiparkinsonia patients and normal controls.

	Group		*P*	Group		*P*
	PD-L	Control		PD-R	Control	
**Single-task**
**PPT scores**
Symptomatic hand	11.97 ± 1.97	14.47 ± 1.36	**<0.001**	12.19 ± 2.09	15.60 ± 1.77	**<0.001**
Asymptomatic hand	14.22 ± 1.59	15.60 ± 1.77	**0.011**	12.83 ± 1.77	14.47 ± 1.36	**<0.001**
**3-subtraction**	11.75 ± 3.45	13.38 ± 4.33	0.180	11.83 ± 3.07	13.38 ± 4.33	0.143
**Dual-task**
**PPT performance**
Symptomatic hand	10.56 ± 1.91	12.48 ± 2.21	**0.005**	9.83 ± 2.39	13.36 ± 2.13	**<0.001**
Asymptomatic hand	11.59 ± 1.96	13.36 ± 2.13	**0.008**	11.19 ± 1.91	12.48 ± 2.21	**0.025**
**3-subtraction**
Symptomatic hand	10.69 ± 2.78	10.98 ± 3.01	0.441	9.29 ± 2.78	11.58 ± 4.14	**0.039**
Asymptomatic hand	10.94 ± 2.36	11.58 ± 4.14	0.630	10.60 ± 2.22	10.98 ± 3.01	0.383
**Interference-effect (PPT performance)**
Symptomatic hand	−11.28%	−13.59%	0.554	−19.32%	−14.18%	0.124
Asymptomatic hand	−17.50%	−14.18%	0.430	−12.16%	−13.59%	0.704
**Interference-effect (3-subtraction)**
Symptomatic hand	−6.58%	−15.69%	0.121	−14.62%	−10.52%	0.461
Asymptomatic hand	−7.98%	−10.52%	0.563	−7.9%	−15.69%	0.116

*PD-R, right-onset Parkinson’s disease; PD-L, left-onset Parkinson’s disease; PPT, Purdue Pegboard Test; The bold emphasis in the p column means *p* < 0.05 with statistically significant*.

### Performance on the PPT and 3-Subtraction Task: Dual-Task Condition

Compared to the single-task condition, adding a concurrent cognitive task to the PPT produced the expected reduction of scores in hand dexterity in all three groups (Supplementary Figure S2). Interestingly, however, the effect of dual-task interference on PPT performance was significantly greater in the PD-R than in that of PD-L group when these two groups of patients used their symptomatic hand (−19.32% vs. −11.28%, *P* = 0.046), although the interference effect was similar between the PD-L/PD-R and normal control groups, for both the symptomatic hand and asymptomatic hand performance. Moreover, compared to the normal control subjects, the cognitive performance was significantly inferior when patients in the PD-R group performed concurrently the pegboard test with their symptomatic hand (*p* = 0.039; Table 2).

### Linear Model and *post hoc* Analysis

With regards to the PPT scores under both single- and dual-task conditions, a linear model involving both age and gender adjustment revealed a significant main effect of groups (PD-L, PD-R and control) as well as a significant difference between conditions (single- vs. dual-task), but no group and condition interaction for the NDLH group (group, *F* = 20.695, *P* < 0.001; condition, *F* = 30.633, *P* < 0.001; interaction, *F* = 0.319, *P* = 0.727). A similar pattern was also found based with the DRH performance (group, *F* = 46.833, *P* < 0.001; condition, *F* = 51.631, *P* < 0.001; interaction, *F* = 0.101, *P* = 0.904). Further *post hoc* analyses revealed that performance on the PPT with the NDLH in both PD-L (*P* < 0.001) and PD-R (*P* < 0.001) was worse than that of the normal control group. Yet most importantly, there was no significant difference between PD-L and PD-R (*P* = 0.080) subgroups, even though performance of the PD-R patients with the NDLH was clinically unaffected, hence suggesting that performance on the PPT could be used to detect motor deficits in the asymptomatic hand of PD-R patients. As expected, performance on PPT with the DRH in the PD-R patients was worse than normal controls as well as PD-L patients (all *P* < 0.001), while we observed a comparable level of performance in the DRH PPT performance between normal controls and PD-L patients, thus suggesting a potential compensatory mechanism underlying the asymptomatic hand (DRH) in PD-L patients.

Results in the 3-subtraction task revealed that the main effect of conditions (single- vs. dual-task) was significantly different when using the NDLH (*P* = 0.007) and DRH (*P* = 0.006), while the main effect of group (PD-L, PD-R, and control) was statistically significant only for the DRH performance. Further *post hoc* analyses showed that performance of the PD-R group was worse than that of the normal controls (main effect *P* = 0.009, *post hoc* *P* = 0.006, respectively).

### Discriminating Hemiparkinsonian Patients From Normal Controls: ROC AUCs of Pegboard Test

**Figure 1:**
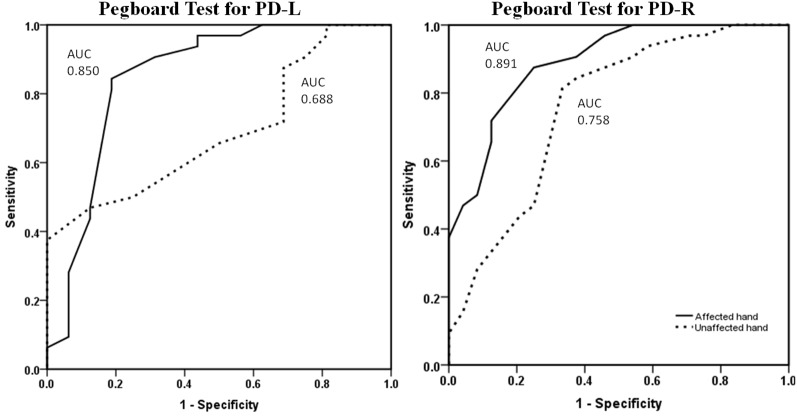
ROC curves for symptomatic and asymptomatic hand PPT performance of PD-L and PD-R patients vs. normal controls. The symptomatic hand (dotted line) and asymptomatic hand (continuous line) pegboard dexterity for discriminating patients and normal controls. ROC, receiver operating characteristics; PPT, Purdue Pegboard Test; PD-L, left-onset Parkinson’s disease; PD-R, right-onset Parkinson’s disease; AUC, area under the ROC curve.

Using a ROC curve analysis approach, the pegboard test revealed reasonable discriminative ability based upon the performance of PD patients compared to normal control subjects (Supplementary Table S2). When looking at the symptomatic hand, PPT scores showed a level of sensitivity above 75% and of specificity above 85% to differentiate from the normal hand. For the asymptomatic hand, the level of pegboard dexterity revealed an AUC of 0.758 for the PD-R group, with relative satisfactory accuracy, and 0.688 for the PD-L group, which is considered modestly accurate for a potential diagnostic test (Figure 1).

### The Correlation Between PPT Scores and Clinical Motor Features in *de novo* Patients With PD

PPT scores in patients with PD were significantly correlated with their UPDRS III motor scores, even when correcting for age, gender and disease duration (*β* = −0.540, *P* < 0.0001; Supplementary Figure S3). While separating the pooled of PD patients in PD-R and PD-L groups, the PPT scores were also negatively correlated with the UPDRS part III and bradykinesia sub-score.

### Association Between PPT Scores in PD Patients and the Time Interval Before Converting to Bilateral Affected Symptoms

**Figure 2:**
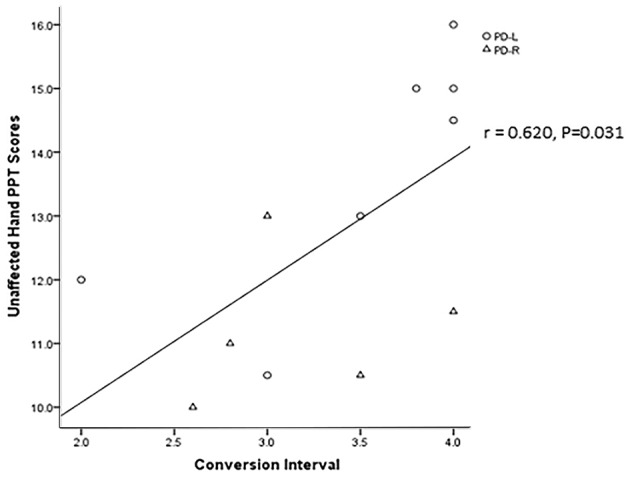
The correlation between the patients’ asymptomatic hands baseline PPT scores and their unilateral affected to bilateral affected conversion interval.

Twenty-five patients with PD (25/40) were contacted over a period of 4 years after baseline through telephone calls. Two of them were excluded because one patient died while the other one could not respond to questions clearly. Among the 23 PD patients included in the final analyses (Supplementary Figure S4), 12 PD patients (7 PD-L, 5 PD-R) converted from a unilateral to a bilateral affected disease condition. Importantly, the baseline performance on the PPT based using the asymptomatic hand of patients in the PD-R group who remained unilaterally affected was significantly higher than that of patients who converted to a bilateral affected condition (13.56 ± 1.36 vs. 11.20 ± 1.15, *P* = 0.007; Supplementary Table S3). Furthermore, a positive correlation between baseline PPT performance of the asymptomatic hands and the time interval before converting conversion interval was observed in PD patients (*N* = 12, *r* = 0.6200, *P* = 0.031; Figure 2).

## Discussion

Our study confirms and extends the results of previous reports that manual dexterity functions are impaired in PD patients, that their performance can be distinguished with relatively high sensitivity and specificity compared to matched control participants, and that their performance on PPT is positively correlated with disease severity as measured using the UPDRS III motor scores (Haaxma et al., 2008, 2010). Moreover, our findings add to those from other studies reports by showing that such a pattern of results can also be observed when strictly unilaterally affected PD patients are performing with their asymptomatic hand. Importantly, however, other novel findings from this study are that the DRH is more sensitive to dual-task interference in the early disease stage, and that PPT performance of the asymptomatic hand in unilaterally affected PD patients is associated with the time interval patients converted from clinically unilateral to bilateral symptomatic cases. These results thus suggest that PPT performance can serve as an objective auxiliary behavior marker that can aid in detecting prodromal stage of PD patients, and in monitoring disease progression (Darweesh et al., 2017).

Two studies have previously investigated the effects of adding a cognitive task while performing the PPT and demonstrated a greater dual-task interference in PD patients compared to normal participants (Proud and Morris, 2010; Zhou et al., 2013). Yet in the latter studies, PD patients were tested when taking their usual medication, and moreover, no detailed neuropsychological evaluation was carried out. In our drug-naive unilaterally affected cases, the dual-task interference seemed greater in PD patients with the dominant right affected hand, while no significant difference between such early PD patients and normal controls on cognitive-motor interference was observed. To our knowledge, this is the first study on dual-task PPT aimed at enhancing detection of PD at prodromal stage, and the results showed that adding the serial subtraction task on PPT did not have added value in differentiating the early clinical cases and matched control participants. The cognitive-motor dual-task impairment in PD is thought to result from dopamine deficiency in the prefrontal-basal ganglia-thalamo-cortical loops, although the precise neural substrate underlying the patients’ deficit depends on the type of tasks administered (Kelly et al., 2012; Schönberger et al., 2015). However, although conjectural, it is likely that the dopaminergic deficit is limited to the sensorimotor territories of the striatum contralateral to the clinically unaffected side, with preserved associative regions of the striatum and cortical connection that assures the intact cognitive-motor performance in “prodromal” patients as compared to normal controls. The sensorimotor and associative regions of the striatum are gradually involved in disease-related progression (Obeso et al., 2014). This is consistent with our previous finding that the “prodromal” patients demonstrate unaffected initial motor sequence learning (Dan et al., 2015). Moreover, while competition for limited resources has usually been proposed to explain dual-task interference, the compensatory mechanism of other brain areas in the very early stage of PD could be another explanation for the comparable dual-task interference effect found between the unilateral PD and normal control participants (Kelly et al., 2012; Tuovinen et al., 2018; Xu et al., 2019).

Based on the fact that patients with PD-R and PD-L were well matched with respect to their UPDRS-III scores, one can assume that the degree of dopaminergic denervation should be similar in the two groups. However, dual-task interference effect showed that the DRH PPT performance in PD-R group was selectively impaired, whereas performance of the PD-L group with the NDLH was similar to that of the normal control group. This indicates and extends previous studies, which have reported that the motor loop or pathway contralateral to the dominant and non-dominant hand might function differently after PD-related dopaminergic loss (Huang et al., 2017). The dominant, better-skilled hand, might be more sensitive to cognitive-motor dual-task after dopamine depletion. Growing evidence suggests that exercise/skill training can induce brain neuroplasticity and synaptic reorganization. However, neuro-computational simulations confirm that motor impairments and pathways imbalances may also result from dysfunctional synaptic plasticity in unmedicated PD (Hirsch and Farley, 2009; Schroll et al., 2014; Abbruzzese et al., 2016). Moreover, other studies have shown movement/practice-related increases of beta modulation in normal subjects but not in PD patients. They concluded that such changes represent saturation of cortical plasticity (Nelson et al., 2017). Taken together, we speculate that the saturation of neuroplasticity and then dysfunctional synaptic plasticity of the contralateral brain to the more skilled dominant hand in PD-R patients may contribute to its fragility during the dual-task test condition, although future studies are needed to test this hypothesis.

As expected, the results of the present study corroborate those of previous investigations, which have also shown the presence of fine motor deficits in the symptomatic hand of PD cases. However, and most importantly, the asymptomatic hand of patients showed the predicted decline in dexterity compared to the corresponding hand of normal controls in both single- and dual-task conditions of the PPT. This suggests that PPT performance can be impaired even before the classical clinical motor symptoms manifest in PD patients. Moreover, PPT’s performance of the asymptomatic hand revealed an AUC around 0.70 for patients (PD-R: 0.758; PD-L: 0.688, respectively) vs. controls, and was associated with conversion from a clinically unilateral to bilateral affected state, indicating that PPT can detect the subclinical fine motor abnormalities in the earliest stage of PD. These results are in line with previous studies, which demonstrated that PPT is a useful instrumental test that can differentiate PD patients from age-matched controls and correlates well with the nigrostriatal dopaminergic degeneration (Vingerhoets et al., 1997; Haaxma et al., 2010; Ruzicka et al., 2016). Therefore, our findings further reveal that the PPT is a sensitive measure to detect PD patients in the prodromal phase, particularly in the NDLH-onset cases (the asymptomatic hand of PD-R).

Although this study shows PPT can serve as a potential behavioral marker in detecting and monitoring disease progression in the very early stage of PD, the present study was not without limitations. First, the asymptomatic side in unilaterally affected PD patients is thought to represent a good model of the “prodromal” stage of the disease. Yet the present study does not provide direct pathological evidence (e.g., neuronal loss measured through positron emission tomography (PET) scanning), although the telephone follow-up provided indirect evidence for the symptomatic conversion. It would thus be important in future studies to use PET confirmation in subjects at high risk for PD. Second, although we observed that PPT was associated with severity and progression of the disease, the sample was small, especially in the follow-up test. Furthermore, only a telephone, not a clinical follow-up assessment was carried out, and thus a validation study with a much larger sample would be necessary. Third, as we did not address the specificity of PPT with respect to other forms of parkinsonism or any other diseases, administering the PPT in isolation as a screening tool in general population risk to produce high numbers of false-positive diagnosis of PD patients, although it has been reported PPT isolation can help to identify high-risk individuals for neurodegenerative disease in community (Darweesh et al., 2017). Thus, it is reasonable to use PPT in combination with other tests battery e.g., genetic tests, non-motor screening tests to improve the sensitivity to identify prodromal PD patients.

In conclusion, this preliminary study demonstrates that impaired PPT performance can be observed in “prodromal phase” of PD and that dual-tasking does not have added value in differentiating between the early clinical cases and normal control subjects. The laterality difference during the PPT dual-task performance may shed light on the mechanism of brain plasticity in the very early disease stage. Further studies with larger sample size and longitudinal clinical interview follow-up are needed, however.

## Data Availability Statement

All datasets generated for this study are included in the manuscript/Supplementary Files.

## Ethics Statement

The studies involving human participants were reviewed and approved by Xuanwu Hospital of Capital Medical University. The patients/participants provided their written informed consent to participate in this study.

## Author Contributions

XD, JD and PC conceived and designed the project. JL, JM and YZ collected the data of subjects and tasks performance. XD and JL analyzed the data. XD, JD and PC wrote and reviewed the manuscript. All authors read and approved the final manuscript.

## Conflict of Interest

The authors declare that the research was conducted in the absence of any commercial or financial relationships that could be construed as a potential conflict of interest.
