# Supplementary material for: Impaired Fine Motor Function of the Asymptomatic Hand in Unilateral Parkinson’s Disease
Source: Front Aging Neurosci. 2019 Oct 4;11:266. doi: 10.3389/fnagi.2019.00266 (PMC6787142; doi:10.3389/fnagi.2019.00266)
Supplement: Supplementary file 1 [file Data_Sheet_1.doc]

**Supplementary Table 1** The normal distribution test for data from the PPT and cognitive test during single-task and dual-task conditions.

|  | **Groups** | **P^a^** | **P^b^** | **Skewness** | **SD** | **Kurtosis** | **SD** |
| --- | --- | --- | --- | --- | --- | --- | --- |
| **Single-task** | | | | | | | |
| **PPT scores** |  |  |  |  |  |  |  |
|  | PD-R | 0.200 | 0.337 | 0.102 | 0.472 | -1.098 | 0.918 |
| NDLH | PD-L | 0.200 | 0.185 | 0.905 | 0.564 | 0.308 | 1.091 |
|  | NC | 0.012 | 0.229 | -0.272 | 0.414 | 0.428 | 0.809 |
|  | PD-R | 0.200 | 0.276 | 0.043 | 0.472 | -1.061 | 0.918 |
| DRH | PD-L | 0.006 | 0.034 | -1.096 | 0.564 | 0.474 | 1.091 |
|  | NC | 0.152 | 0.536 | 0.254 | 0.414 | -0.697 | 0.809 |
| **3 subtraction** | PD-R | 0.188 | 0.590 | -0.112 | 0.472 | -0.840 | 0.918 |
|  | PD-L | 0.200 | 0.203 | 0.951 | 0.564 | 0.421 | 1.091 |
|  | NC | 0.198 | 0.096 | 0.629 | 0.421 | -0.096 | 0.821 |
| **Dual-task** |  |  |  |  |  |  |  |
| **PPT scores** |  |  |  |  |  |  |  |
|  | PD-R | 0.200 | 0.387 | -0.437 | 0.472 | -0.840 | 0.918 |
| NDLH | PD-L | 0.187 | 0.045 | 1.301 | 0.564 | 1.716 | 1.091 |
|  | NC | 0.200 | 0.415 | 0.334 | 0.414 | -0.359 | 0.809 |
|  | PD-R | 0.200 | 0.463 | 0.100 | 0.472 | -0.792 | 0.918 |
| DRH | PD-L | 0.200 | 0.131 | -0.467 | 0.564 | -0.593 | 1.091 |
|  | NC | 0.133 | 0.211 | -0.157 | 0.414 | 0.941 | 0.809 |
| **3 subtraction** | | | | | | | |
|  | PD-R | 0.200 | 0.110 | 0.090 | 0.472 | -1.403 | 0.918 |
| NDLH | PD-L | 0.200 | 0.642 | 0.402 | 0.564 | -0.442 | 1.091 |
|  | NC | 0.001 | 0.089 | 0.338 | 0.414 | 0.857 | 0.809 |
|  | PD-R | 0.152 | 0.481 | 0.336 | 0.472 | -0.720 | 0.918 |
| DRH | PD-L | 0.094 | 0.659 | 0.200 | 0.564 | 0.562 | 1.091 |
|  | NC | 0.200 | 0.766 | -0.239 | 0.421 | 0.092 | 0.821 |

PD-R, right-onset Parkinson's disease; PD-L, left-onset Parkinson's disease; NC, normal control; PPT, Purdue Pegboard Test; NDLH , non-dominant left hand; DRH, dominant right hand; SD, standard deviation.

^a^ p value was calculated from the Kolmogorov-Smirnov test.

^b^ p value was calculated from the Shapiro-Wilk test.

**Supplementary Table 2** AUC for the Purdue Pegboard performance for hemiparkinsonian patients versus controls

for sing-task and dual-task conditions

|  | **ROC/AUC** | **P** | **95%CI** | **Cutoff** | **Sensitivity** | **Specificity** |
| --- | --- | --- | --- | --- | --- | --- |
| **Single-task** |  |  |  |  |  |  |
| **Symptomatic hand** |  |  |  |  |  |  |
| PD-L | 0.850 | 9.0x10^-5^ | 0.714-0.985 | 13.25 | 81.2% | 84.4% |
| PD-R | 0.891 | 6.5x10^-7^ | 0.808-0.975 | 13.75 | 75.0% | 87.5% |
| **Asymptomatic hand** |  |  |  |  |  |  |
| PD-L | 0.688 | 0.035 | 0.538-0.839 | 16.25 | 100% | 37.5% |
| PD-R | 0.758 | 0.001 | 0.627-0.890 | 13.75 | 66.7% | 81.2% |

AUC, area under the ROC curve; ROC, receiver operating characteristics; CI, confidential interval; PD-L,

left-onset Parkinson's disease; PD-R, right-onset Parkinson's disease.

**Supplementary Table 3** The asymptomatic hand PPT scores of patients in the 4-year follow-up

| **Groups** | **Converted to bilateral affected** | | **P^c^** | **P^d^** |
| --- | --- | --- | --- | --- |
|  | **No** | **Yes** |  |  |
| PD-L  (N=9; 2:7)^a^ | 13.25±1.77 | 13.71±1.95 | 0.773 |  |
| PD-R  (N=14; 9:5)^b^ | 13.56±1.36 | 11.20±1.15 | 0.007 | 0.013 |

^a^ 2 patients unconverted to bilateral affected versus 7 patients converted to bilateral affected

^b^ 9 patients unconverted to bilateral affected versus 5 patients converted to bilateral affected

^c^ P value was calculated from the student's t test (since the data is normal distribution)

^d^ P value was calculated from the mann-whitney u test (since the data sample is small)

**Supplementary Figure 1 The Purdue Pegboard test tasks for the participants**

**
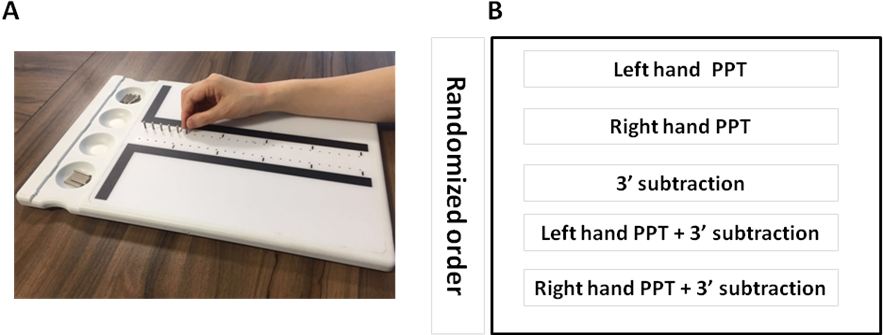
**

PPT: Purdue Pegboard Test

(A) the pegboard test performed with the right hand; (B) the pegboard test performed with right hand and left hand, the cognition test and the dual-task were administered in randomized order. Each test was administrated three trials in a row and each trial was 30 seconds.

**Supplementary Figure 2 Boxplot for PPT performance under both uni-task and dual-task conditions among three groups**


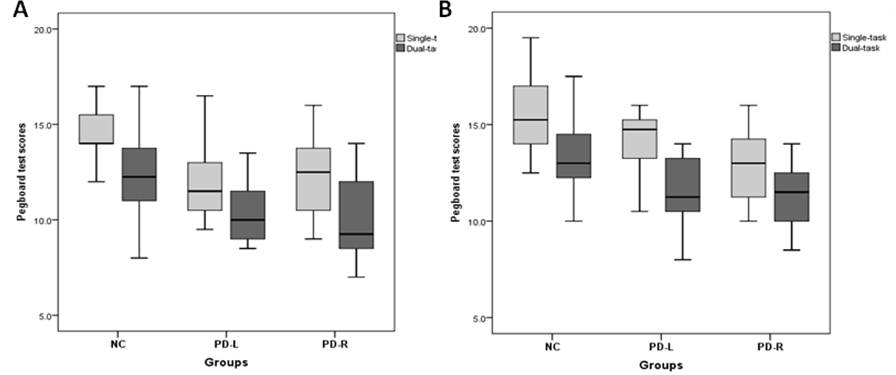


PPT, Purdue Pegboard Test; NC, normal control; PD-L, left-onset Parkinson's disease; PD-R, right-onset Parkinson's disease.

(A) the pegboard test performance of the symptomatic hands of both groups of patients and the left hand of normal controls during the single-task and dual-task condition; (B) the pegboard test performance of the asymptomatic hands of both groups of patients and the right hand of normal controls during the single-task and dual-task conditions.

**Supplementary Figure 3** **Correlation of dual task PPT scores with the UPDRS part III scores**


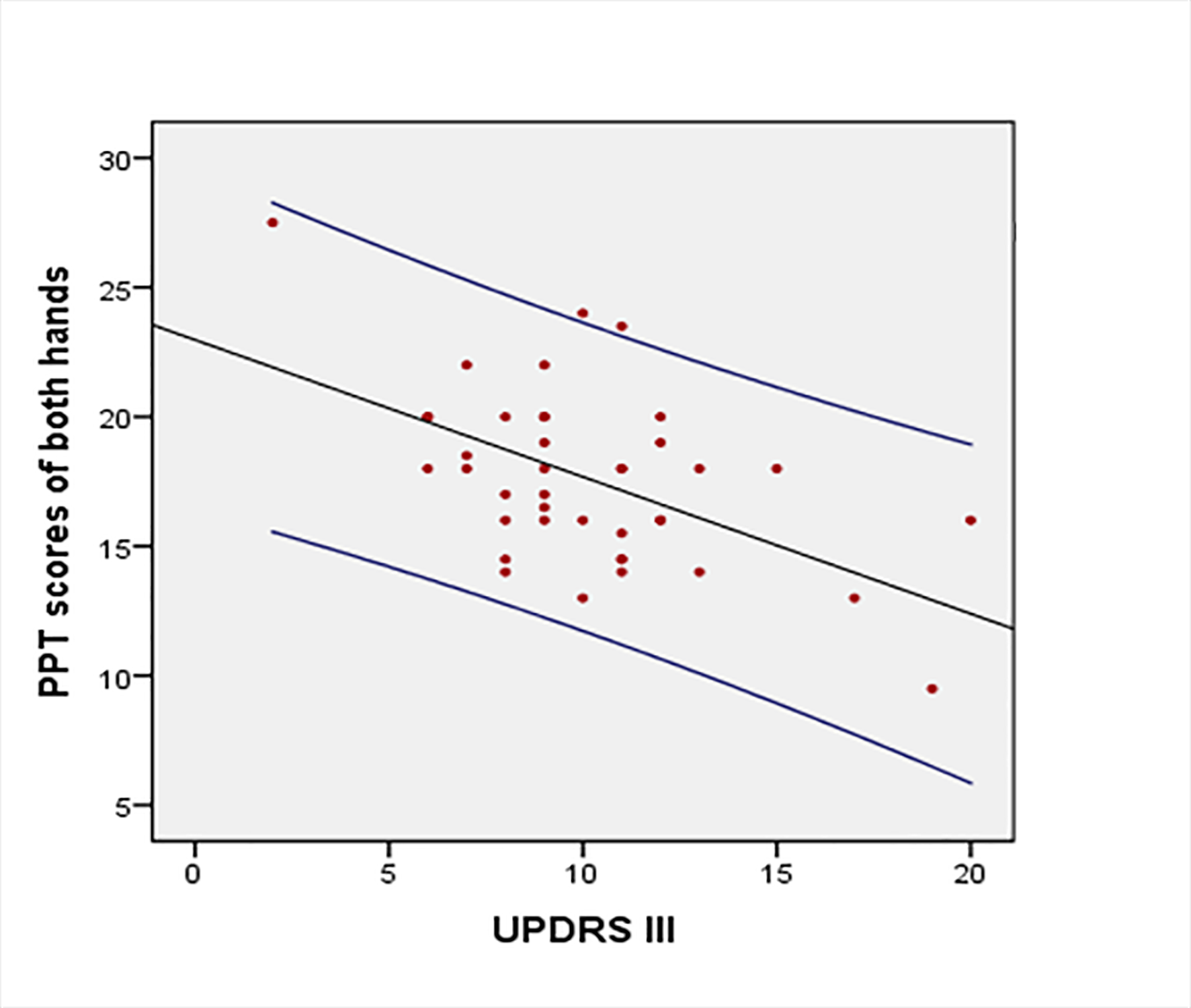


PPT, Purdue Pegboard Test; UPDRS, Unified Parkinson’s Disease Rating Scale.

The PPT scores was the sum of both hands scores when doing the PPT bimanually.

The correlation were calculated under dual-task condition. r=-0.540 p <0.0001

**Supplementary Figure 4 Chat flow of the 4-year follow up for the patients of PD in this study**

**
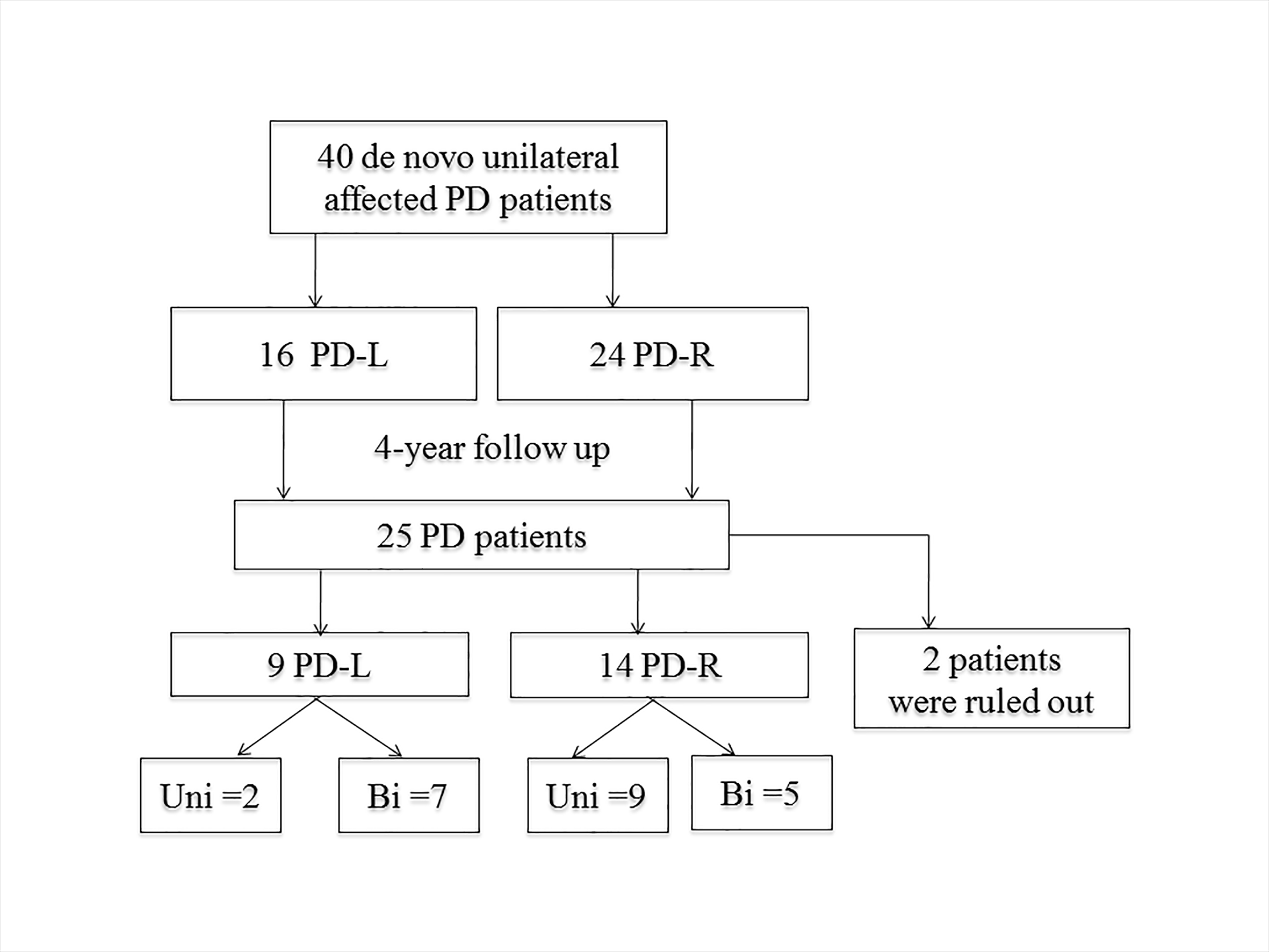
**

PD-L, left-onset Parkinson's disease; PD-R, right-onset Parkinson's disease;

Uni: unilateral affected; Bi: bilateral affected
